# Blackwater fever in Congolese children: a report of clinical, laboratory features and risk factors

**DOI:** 10.1186/1475-2875-12-205

**Published:** 2013-06-15

**Authors:** Joseph M Bodi, Célestin N Nsibu, Roland L Longenge, Michel N Aloni, Pierre Z Akilimali, Pierre M Tshibassu, Patrick K Kayembe, Ahmeddin H Omar, Kenji Hirayama, Jan Verhaegen

**Affiliations:** 1 Department of Paediatrics, Paediatric Emergency and Intensive Care Unit, University Hospital of Kinshasa, School of Medicine, University of Kinshasa, Kinshasa, Democratic Republic of Congo; 2 Department of Paediatrics, Division of Haemato-oncology and Pediatric Nephrology, University Hospital of Kinshasa, School of Medicine, University of Kinshasa, Kinshasa, Democratic Republic of Congo; 3 Division of Biostatistics and Epidemiology, School of Public Health, University of Kinshasa, Kinshasa, Democratic Republic of Congo; 4 Department of Paediatrics, Paediatric Gastroenterology and neurology Unit, University Hospital of Kinshasa, School of Medicine, University of Kinshasa, Kinshasa, Democratic Republic of Congo; 5 Division of Malaria Control (DOMC), Ministry of Health, Nairobi, Kenya; 6 Department of Immunogenetics, Institute of Tropical Medicine (Nekken), University of Nagasaki, Tokyo, Japan; 7 Department of Microbiology, Katholieke Universiteit de Leuven, Brussels, Belgium

**Keywords:** Blackwater fever, Children, Quinine ingestion, Parasitaemia, Kinshasa, Democratic Republic of Congo

## Abstract

**Background:**

Blackwater fever (BWF) is one of the severe forms of malaria. This complication was first described among non-immune European expatriates in the malaria endemic areas. Recently, resurgence of this form of malaria has been reported among the indigenous populations. The objective of this study was to investigate the risk factors among BWF patients.

**Methods:**

A case–control study was conducted between in four hospitals located in Kinshasa, Democratic Republic of Congo from January 2010 to December 2011. One hundred and twenty nine children were recruited with 43 (cases) and 86 (control).

**Results:**

No significant difference in the gender and age distribution was observed between the case and control). The sex-ratio male to female in the case group and control group was respectively 1:1.0 and 1:1.1. The mean age was 8.62 years (SD = 3.84) in patients with haemoglobinuria and 8.55 years (SD = 3.77) in the control group. No difference in frequency of co-infection with *Plasmodium falciparum* and *Plasmodium malariae* was observed between the two groups. Significant differences in haemoglobin, haematocrit, creatinine, urea and platelets levels were observed between the two groups (p < 0.001), but not for blood group and lactate dehydrogenase (LDH) level. Majority of the BWF cases occurred during the rainy season (88.4%). Treatment with quinine (95.3%) was significantly associated with cases (p < 0.001). Seven (16.2%) of the haemoglobinuric children developed acute renal failure.

**Conclusion:**

Rainy season, low parasitaemia and quinine ingestion were the major risk factors significantly associated with haemoglobinuria. Acute renal failure was observed as the major complication of BWF.

## Background

Despite tremendous improvement in control measures in the last decade, malaria still remains a major public health problem in the world, with sub-Saharan Africa accounting for 81% of the 216 millions of malaria cases reported in the world in 2010 [1]. According to World Health Organization (WHO) global estimations in 2010, 655.000 deaths occurred and 86% of them among children less than five years old [1]. *Plasmodium falciparum* is the main species associated with complicated malaria cases such as blackwater fever (BWF) [2].

BWF is a clinical syndrome characterized by an acute intravascular haemolysis presenting with dark urine (haemoglobinuria), jaundice, anaemia and fever. Abdominal pain, vomiting, hepatomegaly, splenomegaly, pain in the back or limbs, dyspnea, tachycardia, generalized malaise and dizziness can also be found. However, patients can also be asymptomatic [3,4]. The ingestion of quinine by non-immune residents affected with *P. falciparum* in endemic areas was associated with BWF [5-9]. Isolated cases of BWF have also been reported from patients treated with other anti-malarial drugs, such as halofantrine, mefloquine and artemether-lumefantrine [10-13].

BWF was first described in non-immune European expatriates who lived in endemic malaria regions [5,6,14-18]. Rare in indigenous populations, the first cases have been reported in Southeast Asia [2] and among African children in Senegal [19]. Recently, resurgence of this disease was reported in other African populations living in areas with stable malaria transmission who are supposed to have sufficient immunity against malaria [3,19-22]. In the Democratic Republic of Congo (DRC), BWF caused acute renal failure in children with more than 22% of mortality rate [23,24].

The pathogenesis of BWF is complex. Inadequate malarial immunity, misuse of quinine and G6PD deficiency have been associated with the occurrence of BWF [5-9]. G6PD status was already studied in DRC where the prevalence varies from 6.1% to 23.4% [25].

The objective of this study was to investigate and determine the risk factors associated with BWF among children with malaria in DRC. The epidemiological, immunological and genetic characteristics of BWF patients was investigated in comparison to those with uncomplicated malaria.

## Methods

### Subjects, study design and case definitions

This case–control study was conducted in four paediatric health facilities in Kinshasa, namely Pediatrics Department of Kinshasa University Hospital, Kimbanseke Hospital, Bondeko Hospital and General Provincial Hospital of Kinshasa. Each of these health facilities represents a stratum of the four administrative subdivisions of Kinshasa, a town of at least 8 million of inhabitants located in an endemic malaria area with a stable transmission. Kinshasa, the capital of DRC, is characterized by two distinct seasons, a rainy season with an average of 1350 mm of annual rainfall during the months of September to May and a hot dry season from June to August.

Children with BWF were recruited as cases, while those with uncomplicated malaria according to WHO definition [1] were enrolled as the control in the study. For each case, two control patients matched for age, sex and place of residence were recruited into the study. The inclusion criteria for BWF cases selection included presence of haemoglobin in the dark urine of febrile patients with jaundice and anemic among patient with microscopically confirmed *P. falciparum* malaria.

### Data collection procedure and blood analysis

After obtaining an informed consent from the parent, each child’s caregiver was interviewed using a structured standard project questionnaire to obtain detailed history of the participants disease or from the child if old he/she enough. A complete physical examination was carried out on each child with focus on detection of anemia, jaundice, hepatomegaly and splenomegaly. Five ml of venous blood sample was drawn from each study participant into an EDTA tube, used to determine haemoglobin, haematocrit, haemoglobin electrophoresis, G6PD screening, blood group, alanine aminotransferase (ALAT), aspartate aminotransferase (ASAT), lactate dehydrogenase (LDH), bilirubin levels, urea and creatinine. Packed cell volume (PCV) was determined using the capillary method and spinning in a Hawksley micro-haematocrit centrifuge at 11,000 g for 5 minutes at room temperature. The PCV was then read with a haematocrit reader.

Thick and thin films were fixed with Giemsa for malaria parasite screening and identification. All participants in the study were screened for Sickle cell trait and glucose-6-phosphate dehydrogenase deficiency (G6PD). G6PD was determined using the fluorescent spot test method of Beutler and Mitchell (Biolabo, france) [26]. For the control group, G6PD was screened at admission but for the case group, G6PD was screened three months after the acute haemolysis crisis.

### Urine collection procedure and urinalysis

All participants were requested to give 20 ml of urine at recruited time and this was then assessed for haemoglobinuria using three test strips. The Test strips used in this study were; Medi Test Combi 9® (Macherey Nagel, Germany), the Urine 9 parameters Combur Test (Cypress Diagnostics, Belgium) and the Urine 9 parameters (Cypress Diagnostics, Belgium), and the results obtained using this strips was compared with the spectrophotometer results (the gold standard). The test strips were dipped into 20 ml of fresh urine sample for approximately one second, then drawn across the rim of the container to remove the excess urine. After 30 to 60 seconds, the strip was assessed using the colour scale, with the test being considered positive if the colour changed from yellow to green. Renal failure was defined as urine output < 0.5 ml/kg for 24 h or a persisting high plasma creatinine concentration above the age-related normal range, after rehydration [27].

### Data management and analysis

All data obtained from the study questionnaire, clinical results were manually entered into a microcomputer and analysed using the Epi-Info 7 Version 2002 (CDC). After data cleaning (control for quality and coherence), they were exported on SPSS 18.0 for further analysis. Descriptive analysis were used to get mean for quantitative and proportion for all the qualitative variables. The confidence interval at 95% was calculated. Associations between variables and haemoglobinuria was evaluated using chi-square and fisher exact test (for the cell with expected frequency less than 5 in two by two table more than 20%). The comparison of continuous variables was calculated using the Student T test. Logistic regression analysis was used to identify determinants factor of BWF occurrence and to measure the strength of association of each determinants (adjusted odds ratio). Statistical significance level was set at p = 0.05.

### Ethical consideration

Ethical approval for the study was granted by the Ethical Committee of the Public Health School of the University of Kinshasa, Kinshasa, DRC (ESP/CE/027B/2011) and the institutional review committee of Institute of Tropical Medicine (NEKKEN), Nagasaki University, Japan. A written consent signed by parents was obtained from each of the subjects recruited before their inclusion.

## Results

A total of 129 children, 43 with haemoglobinuria and 86 with uncomplicated malaria were recruited into the study over the 24 months (January 2010 to December 2011) of the study period. In general, the study participants comprises 68 (52.7%) females and 61 (47.3%) males with a male to female sex-ratio of 1:1.1 and the age ranging from 2 to 15 years with a mean age of 8.57 (SD = 3.73) years (Table 1). The mean age of the patients with haemoglobinuria was 8.62 (SD = 3.84) years while that of the uncomplicated malaria was 8.55 (SD = 3.77) years (Table 1). The sex-ratio male to female in the case group and control group was respectively 1:1.0 and 1:1.1. No significant difference in the gender and age distribution was observed between the case and control.

**Table 1:** Socio-demographic, epidemiological characteristics and malaria parasitaemia among study children and episode of haemoglobinuria

	**Haemoglobinuria**				
	**Positive n (%)**	**Negative n (%)**	**All n (%)**	**OR (IC95%)**	**p**
**Age distribution**					NS
- ≤ 5 ans	8(18.6)	20(23.3)	28(21.7)	1	
- > 5 ans	35(81.4)	66(76.7)	101(78.3)	1.33 (0.53–3.32)	
**Sex. n**					NS
- Male	21(48.8)	40(46.7)	61(47.3)	1.10 (0.53–2.28)	
- Female	22(51.2)	46(53.5)	68(52.7)	1	
**G6PD Status (UI/L)**					0.017
Normal	35(81.4)	52(60.5)	87(67.4)	2.86 (1.86–6.91)	
Deficiency	8(18.6)	34(39.5)	42(32.6)	1	
**LDH (UI/L)**					NS
< 200	20(46.5)	40(46.5)	60(46.5)	1.29 (0.57–2.91)	
200 à 400	14(32.6)	36(41.9)	50(38.8)	1	
> 400	9(20.9)	10(11.6)	19(14.7)	2.31 (0.78–6.89)	
**Blood group**					NS
A	7(16.3)	23(26.8)	30(23.3)	0.57 (0.22–1.52)	
AB	3(7.0)	6(7.0)	9(7.0)	0.94 (0.14–4.87)	
B	8(18.6)	10(11.6)	18(14.0)	1.50 (0.53–4.29)	
O	25(58.1)	47(54.6)	72(55.7)	1	
**Season**					< 0.001
Rainy	38(88.4)	51(59.3)	89(69.0)	5.22 (1.87–14.56)	
Dry	5(11.6)	35(40.7)	40(31.0)	1	
**Plasmodium**					NS
Falciparum	37(86.0)	73(84.9)	110(85.3)	1.10 (0.39–3.12)	
Falciparum and malariae	6(14.0)	13(15.1)	19(14.7)	1	
**Parasitaemia (parasites/microliter)**					0.005
Low	32(78.0)	43(51.8)	76(61.3)	3.31 (1.41–7.78)	
High	9(22.0)	40(48.2)	48(38.7)	1	

Infection was analysed for the case and control groups. Mono-infection with *P. falciparum* was observed in 37 cases (86%) of haemoglobinuric patients, while six cases were observed to have co-infection of *P. falciparum* and *Plasmodium malariae* (14%). Among the control group (uncomplicated malaria cases), 73 children (84.9%) were observed to have mono-infection with *P. falciparum,* while 13 children (15.1%) were observed to have co-infection of *P. falciparum* and *P. malariae*. The mean parasite counts of patients with haemoglobinuria were slightly lower than those uncomplicated malaria with statistically significant difference observed (OR = 3.31(1.41–7.78), p < 0.05) (Table 1).

Distribution of blood group was analysed among the study participants. Even though 55.7% of the study participants were blood group O, no significant difference in the distribution was observed between the case and control groups (58.1.8% vs 54.6%) (Table 1). Distribution of LDH levels as examined on admission was also analysed and no significant difference was observed between the two groups (0R = 2.31 (0.78–6.21), p > 0.05) (Table 1). When the levels of haemoglobin, haematocrit and creatinine levels was compared, it was observed that decreased haemoglobin and haematocrit was significantly associated with increased risk for haemoglobinuria with an odds ratio of [OR: 25.08 (9.54–65.96)] and [OR: 25.54 (9.54–65.96)], respectively (Table 2). On the other hand, it was observed that an elevated creatinine and urea levels were significantly associated with increased risk of haemoglobinuria, with with an odds ratio of [OR: 16.17 (1.97–735.3)] and [OR: 9.6 (3.21–28.68)] (p < 0.001), respectively (Table 2). Urea to creatinine ratio for all the cases was > 1:20 indicating prerenal failure due to dehydration or shock. No case urea to creatinine ratio <1:10 which indicates intrarenal kidney injury that should require renal replacement treatment.

**Table 2:** Laboratory features in study population

	**Haemoglobinuria**				
	**Positive (n = 43)**	**Negative (n = 86)**	**All (n = 129)**	**OR (IC95%)**	**P**
**Haemoglobin (g/dl)**					< 0.001
• patholigical (< 10)	33(76.7)	10(11.6)	43(33.3)	25.08 (9.54–65.96)	
• normal	10(23.3)	76(88.4)	86(66.7)	1	
**Haematocrit (%)**					< 0.001
• pathological (<30)	33(76.7)	10(11.6)	43(33.3)	25.08 (9.54–65.96)	
• normal	10(23.3)	76(88.4)	86(66.7)	1	
**Platelets (number/mm**^**3**^**)**					NS
• pathological	3(7.0)	5(5.8)	8(6.2)	1.21 (0.18–6.60)	
• normal	40(93.0)	81(94.2)	121(93.8)	1	
**Urea(mmol/L)**					< 0.001
• pathological	16(37.2)	5(5.8)	21(16.3)	9.6 (3.21–28.68)	
• normal	27(62.8)	81(94.2)	108(83.7)	1	
**Creatinine (mmol/L)**					< 0.001
• pathological	7(16.3)	0(0)	7(5.5)	16.17 (1.97–753.3)	
• normal	36(83.7)	85(100)	121(94.5)	1	
**Total bilirubin (mg/dl)**					0.011
• pathological	12(27.9)	9(10.5)	21(16.3)	3.31 (1.27–8.65)	
• normal	31(72.1)	77(89.5)	108(83.7)	1	

The majority of haemoglobinuria cases (BWF) occurred during the rainy season (88.4%) [OR = 5.22 (1.87–14.20)], p <0.001), while 59.3%, of the uncomplicated malaria group occurred during the same season (Table 1). On examination the anti-malarial drug taken before among the haemoglobinuria group, 95.3% of the cases were observed to have taken quinine [OR: 50.19 (10.75–234.42)] p < 0.001), while only three patients (4.7%) were observed to have taken ACTs in the group (Table 3).

**Table 3:** Anti-malarials, herbal plants and food intake before episode of haemoglobinuria

	**Haemoglobinuria**				
	**Positive (n = 43)**	**Negative (n = 86)**	**All (n = 129)**	**OR (IC95%)**	**p**
**Anti-malarials**					< 0.001
ACT	2(4.7)	60(69.8)	62(48.1)	1	
quinine	41(95.3)	26(30.2)	67(51.9)	47.31 (10.64–210.3)	
**herbal plants**					0.515
no	41(95.3)	85(98.8)	126(97.7)	1	
yes	2(4.7)	1(1.2)	3(2.3)	4.15 (0.37–47.5)	
**Bean**					0.01
no	37(86.0)	86(100)	123(95.3)	1	
yes	6(14.0)	0(0)	6(4.7)	13.95 (1.62–119.9)	

* ACT Artemisinin-based Combination Therapy.

In this series, it was observed that G6PD activity was significantly low in control group [OR: 0.39 (0 12–0.1.27), p = 0.015] (Table 4). The most common presenting symptoms for the children with haemoglobinuria was observed to be dark urine (100%), jaundice (88.4%), pallor (81.4%) and fever (72.1%), while that for the control group was observed to be 0%, 2.21%, 3.3%, and 100% (p < 0.001). Logistic regression analysis (Table 4) showed that ingestion of quinine, low parasitaemia and rainy season were the main risk factors associated with the haemoglobinuria group (BWF).

**Table 4:** Determinant factors of Black water fever by logistic regression

	**Crude OR (95%CI)**	**p**	**Adjusted OR (95%CI)**	**P**
**Anti-malarials**		< 0,001		< 0,001
• ACT	1		1	
• Quinine	47,31(10,64–210,3)		50,19(10,75–234,42)	
**G6PD status**		0,017		0,115
• Normal (≥276 UI/L)	1		1	
• Deficiency (<276 UI/L)	0,35(0,14–0,54)		0,39(0,12–1,27)	
**Parasitaemia (parasite/microl)**		0,005		0,012
• Low	1		1	
• High	0,30(0,13–0,71)		0,25(0,08–0,74)	

* ACT Artemisinin-based Combination Therapy.

Among the haemoglobinuria patients, seven children (16.2%) developed acute renal failure (ARF), but only two children had peritoneal dialysis performed because of financial constraints. A conservative treatment was carried out for the remaining patients, with three of them developing anuric acute renal failure and succumbing with this complication (6.9%).

## Discussion

In DRC, malaria is endemic, characterized by a high and perennial transmission. However, there are few reports on severe childhood malaria especially BWF. In the present study, the clinical and biological observations and the risk factors of BWF were evaluated for the first time in Central Africa. This case–control study has matched cases and controls for age, sex and location area. It suggests that haemoglobinuria in severe malaria is frequent in children above five years of age. The mean age of these patients in this series was 8.62 (SD = 3.84) years. These findings are comparable with previous studies, which reported the high incidence of BWF in this age even in endemic stable areas where malaria immunity is acquired after six years [3,19,28]. In Nigeria, Jelliffe reported that this syndrome was three times prevalent among children than adults, an observation which shows an evidence of the delay in the acquisition of malarial immunity [29].

When BWF occurs among sufficient acquired malaria immunity patients, some questions must be addressed about the pathogenesis of this severe form of malaria and the immunity status of children who develop haemoglobinuria. The present study shows that children with haemoglobinuria have low parasitaemia compared to those without haemoglobinuria, [OR: 3.31 (1.41–7.78) with p = 0.005]. Many authors support that parasitaemia is low in BWF and sometimes may be absent [1,2,30]. Therefore, it is likely that the parasitaemia alone could not explain the acute intravascular haemolysis which results in haemoglobinuria. Other authors reported high incidence of haemoglobinuria in areas of high malaria transmission focusing on the coexistence of the parasitaemia and level of transmission [29]. This hypothesis has not been confirmed by subsequent studies, such as those carried out in Uganda, where no significant difference was found between the areas of different level of malaria transmission [30,31]. Other risk factors should be explored and may be targets of new investigations to search reasons why acute haemolysis occurs in low parasitaemia patients and in low malaria transmission. This finding supports previous reports showing that BWF is more prevalent in the malaria endemic area and during the intense malaria transmission period [2,32]. It is possible that parasitaemia alone could not explain acute intravascular haemolysis that leads to haemoglobinuria in BWF cases and thus other risk factors including immunological and genetics factors should be investigated to elucidate reasons why acute haemolysis occurs among patients with low parasitaemia patients and in areas with low malaria transmission. The high pretreatment levels can explain low parasitaemia observed during BWF. BWF may be an acquired immune syndrome and the role of immunity should be elucidated in the next step of the study.

*Plasmodium falciparum* remains the most prevalent species associated with the occurrence of severe malaria [2]. In this study, 86% of cases (BWF) were observed to be infected by *P. falciparum* as mono-infection, while 14% had mixed infection of *P. falciparum* and *P. malariae. Plasmodium malariae* is known to cause kidney damage [33]. It is possible that sudden onset of infection could have resulted in renal disturbance among *P. falciparum* infected children.

In this study, it was observed that there was high frequency of G6PD deficiency (39.5%) among the control group compared with the cases (18.6%) [OR = 2.86. (1.86–6.91) p = 0.012] in bi-variate analysis. This enzymatic defect is a known major risk factor for the occurrence of haemoglobinuria (BWF). This often occurs after taking oxidant products like quinine, mefloquine and others. Applying the logistic regression, results show OR: 0.39 (0.12–1.27). The high frequency of G6PD can suggest that G6PD may be high in Congolese population [26] and needs a screening programme to prevent an acute intravascular haemolysis leading sometimes to renal failure. G6PD genotyping should be necessary to get more accurate information about genetics data of this defect in malaria in this settings.

In addition, the results of this study show a significant association between quinine ingestion and the occurrence of haemoglobinuria [OR: 50.19 (10.75–234.42) p < 0.001]. This is in line with previous studies that have reported repeated exposure to quinine as a risk factor for BWF [2,3,19]. Thus, quinine should be avoided in the treatment of uncomplicated malaria.

In this study, no significant difference was in the level of LDH, an enzyme also implicated in the genesis of vascular haemolysis, was observed between the two groups. Since the advent of dialysis, the mortality rate for anuric acute renal failure (AARF) has changed [13]. The access to dialysis is, however, limited in many low-resource countries as is the cases in the DR Congo. All haemoglobinuric patients who developed AARF died because peritoneal dialysis was not available or affordable. This contributes to the high mortality rate of the BWF reported in the DR Congo [23,24]. Furthermore, no significant difference was found between the two groups in the LDH assay in this study, an enzyme also implicated in the genesis of vascular haemo lysis.

A seasonality of hyperhaemolysis attacks was observed in the present series. Haemoglobinuria cases occurred mostly in rainy season than in dry season. This finding is supported by some authors who reported that BWF is more prevalent in the malaria endemic area and during the intense malaria transmission period [2].

Jaundice and pallor manifestations were significantly associated with haemoglobinuria, which is in accordance with previous studies on BWF [2,3,19]. Other complains like oliguria, seizures, comatose, lethargy, dyspnea and lumbar pain also was significant association with haemoglobinuria in this study (p < 0.001); low levels of haemoglobin and packed cell volume (haematocrit) was more frequent among the haemoglobinuric patients. These results confirm the acute intravascular haemolysis resulting in haemoglobinuria and acute renal failure [4].

### Limitations of the study

The design could have been improved by recruitment of a severe malaria cohort without haemoglobinuria. Since, some of the factors such quinine use and rainy season would be less relevant of compared to this group. This design leads to another next study. Variables such anthropometric parameters and cardiac outcome have not been investigated.

## Conclusion

Rainy season [OR: 5.22 (1.87–14.46) p < 0.001], a low parasitaemia [OR: 0.25 (0.08–0.74), p < 0.05 ] and the ingestion of quinine [OR: 50.39 (10.75–234.42), p < 0.001] were the major risk factors associated with haemoglobinuria in this study. Among children with severe malaria, jaundice, severe anaemia and acute renal failure with elevated creatinine were the most common complications of BWF observed in the acute intravascular haemolysis compared to uncomplicated malaria. Immunology and genetics factors should be investigated to get more information about the risk factor of BWF in indigenous population in the endemic malaria areas.

## Competing interests

The authors declare that they have no competing interests.

## Authors’ contributions

CNN, PMT, MNA and JMB, conceived, designed, deployed and directed the case–control study at the Department of Paediatrics at Kinshasa university hospital and wrote the manuscript. RLL carried out patient recruitment and follow-up, sample collection, storage and transport. KI and JV brought some precious corrections. PKK and PPA analysed data. AO edited the English and made corrections. All authors read and approved the final manuscript.
